# NeuroMark-DyFICA: NeuroMark dynamic frequency-informed ICA with high-frequency spatial filtering for windowed fMRI analysis

**DOI:** 10.1162/NETN.a.551

**Published:** 2026-07-20

**Authors:** Neda Behzadfar, Armin Iraji, Najme Soleimani, Tulay Adali, Vince Calhoun

**Affiliations:** Digital Processing and Machine Vision Research Center, Na.C., Islamic Azad University, Najafabad, Iran; Tri-Institutional Center for Translational Research in Neuroimaging and Data Science (TReNDS), Georgia State University, Georgia Institute of Technology, Emory University, Atlanta, GA, USA; Department of Computer Science and Electrical Engineering, University of Maryland, Baltimore County, Baltimore, MD, USA

**Keywords:** Brain networks, Dynamic functional connectivity, fMRI, Independent component analysis (ICA), Schizophrenia, Spatial dynamics

## Abstract

Fine-scale spatial dynamics within functional brain networks manifest as high spatial-frequency variations that conventional independent component analysis (ICA) methods fail to capture. These subtle changes may carry critical information about transient connectivity and disordered brain function. We developed NeuroMark-DyFICA (dynamic frequency-informed ICA), a novel framework to enhance detection of spatiotemporal variability in fMRI data. It integrates three stages: dynamic NeuroMark ICA across sliding windows to estimate time-varying, spatially constrained networks; high-pass spatial filtering to emphasize fine-scale spatial features; and group-level ICA to extract refined dynamic components with subject-specific mixing weights. Unlike prior NeuroMark applications or conventional dynamic ICA, NeuroMark-DyFICA establishes a reproducible latent space of high-frequency dynamics, uniquely capturing transient, fine-scale reconfigurations of network topography. Validation using a controlled 2D simulation demonstrated reliable detection of subtle spatial shifts mimicking pathology, which conventional ICA failed to recover. Applying to resting-state fMRI from schizophrenia patients and healthy controls, multiple networks were estimated. We highlight six representative systems (thalamus, auditory, visual/fusiform, middle frontal, default mode, cerebellum). Results revealed two complementary abnormalities in schizophrenia: an imbalance between inactive and hyper-engaged states and altered convergence among dynamic states. NeuroMark-DyFICA reveals fine-grained spatiotemporal disruptions in brain networks, offering mechanistic insights and potential biomarkers for psychiatric disorders.

## Author Summary

Brain network dynamics are most often studied as changes in the expression or interaction of large-scale, canonical patterns over time. While this perspective has been highly productive, it implicitly emphasizes low spatial-frequency variations and may overlook more focal, transient reconfigurations that occur within otherwise stable networks. Neurophysiological and clinical evidence increasingly suggests that cognition- and disease-related alterations can manifest as subtle, localized shifts in network boundaries, internal structure, or spatial coherence—features that reside in the high spatial-frequency domain and are difficult to capture with conventional dynamic approaches. In this study, we introduce NeuroMark-DyFICA (dynamic frequency-informed independent component analysis), a framework designed to capture dynamic, fine-scale spatial variations in functional brain networks while preserving biologically meaningful network structure. The method integrates template-guided independent component analysis with spatial-frequency filtering and group-level decomposition, enabling isolation of transient, high-frequency spatial changes within canonical networks in a fully automated and reproducible manner. Using controlled simulations, we demonstrate that NeuroMark-DyFICA is sensitive to small spatial shifts that conventional static or low-frequency–focused methods often overlook. When applied to resting-state fMRI data from individuals with schizophrenia and healthy controls, the framework reveals abnormal dynamic spatial patterns across auditory, visual, frontal, default-mode, and cerebellar networks, including altered prevalence and coordination of dynamic spatial states. By explicitly targeting fine-scale spatial dynamics, NeuroMark-DyFICA provides a new lens for studying how brain networks reconfigure over time and how these processes are disrupted in neuropsychiatric disorders. This approach offers a principled pathway toward more sensitive biomarkers and advances precision neuroscience by bridging large-scale network organization with localized, clinically relevant variability.

## INTRODUCTION

Functional brain networks are inherently dynamic, reconfiguring over short timescales in response to both intrinsic neural fluctuations and behavioral demands. Evidence from resting-state and task-based functional magnetic resonance imaging (fMRI) shows that networks transition between different configurations, reflecting not only temporal fluctuations in connectivity but also spatial reorganization within canonical systems (Calhoun & de Lacy, 2017; Fox & Raichle, 2007; Lurie et al., 2020). These small-scale variations—sometimes termed the spatial chronnectome—are thought to support cognitive flexibility and adaptive information processing, while their disruption has been linked to neuropsychiatric disorders such as schizophrenia (SZ), depression, and autism (Bostami et al., 2025; Iraji et al., 2024; Iraji, Deramus, et al., 2019; Iraji, Fu, et al., 2019). Capturing these dynamics is therefore essential for understanding both healthy brain function and disease-related abnormalities.

Despite this importance, most analytic frameworks for fMRI continue to emphasize static or large-scale properties of brain networks. Traditional independent component analysis (ICA), for example, is typically applied in a spatially static fashion, estimating a single set of spatial maps and associated time courses over the entire scan. Such approaches are limited in their ability to capture the dynamic and transient nature of brain connectivity (Iraji et al., 2020; Nielsen et al., 2016). A growing body of work shows that functional networks reconfigure over short timescales, reflecting both intrinsic neural processes and behaviorally relevant states (Iraji, Deramus, et al., 2019; Iraji, Fu, et al., 2019; Iraji et al., 2020), meaning that static ICA risks missing meaningful fluctuations and fine-scale shifts in network organization that may vary across individuals and clinical groups.

To overcome these limitations, several dynamic methods have been introduced. Sliding-window correlation and related temporal approaches have improved sensitivity to time-varying connectivity, revealing transient fluctuations in network coupling (Fong et al., 2019; Iraji et al., 2024; Phadikar et al., 2025; Pusuluri et al., 2024). More recently, extensions of ICA have enabled the characterization of spatial dynamics, capturing reconfigurations in network topographies and subtle spatial shifts within canonical systems (Iraji et al., 2024; Iraji, Deramus, et al., 2019; Iraji, Fu, et al., 2019). These advances have opened the way to investigating the spatial chronnectome, offering new insights into how spatially varying brain states support cognition and how their disruption may underlie psychiatric and neurological disorders (Calhoun & de Lacy, 2017; Iraji, Deramus, et al., 2019). However, most existing dynamic frameworks—commonly referred to as dynamic functional network connectivity (dFNC)—still focus primarily on temporal covariance changes rather than explicit spatial reorganization. Consequently, the fine-scale spatial dynamics that underlie rapid network reconfiguration remain insufficiently captured and rarely modeled directly.

However, challenges remain in scaling data-driven ICA methods in a way that preserves reproducibility across diverse datasets while also providing cross-subject correspondence of the resulting components. To address these issues, the NeuroMark framework was introduced as a hybrid approach which applies spatial priors derived from multiple large independent datasets to guide ICA decomposition in a fully automated and reproducible manner (Du et al., 2020). These spatial priors were established by performing group ICA on large, independent resting-state fMRI cohorts comprising thousands of healthy subjects, followed by replication and stability analyses to identify intrinsic connectivity networks that are consistently expressed across populations and imaging conditions. The resulting templates represent reproducible large-scale brain networks and define a standardized latent space that enables automated labeling and reliable cross-subject correspondence.

By incorporating these priors as soft spatial constraints rather than fixed templates, NeuroMark balances biological interpretability with data adaptivity, allowing subject- and time-specific deviations from the normative patterns to be captured. At the same time, reliance on spatial priors may bias estimation toward canonical network structures, potentially reducing sensitivity to entirely novel or rare configurations; this limitation motivates complementary strategies that explicitly emphasize fine-scale deviations from the normative spatial priors. NeuroMark has demonstrated its utility in identifying reliable biomarkers of brain disorders across multiple cohorts, highlighting the importance of combining data-driven methods with biologically informed constraints.

Building on this foundation, recent work has emphasized the importance of capturing fine-scale spatial variations in brain networks. Our earlier NeuroMark-HiFi framework focused on static fMRI data and demonstrated that small but systematic spatial variations in network topography carry important information for distinguishing individual- and group-level differences (Behzadfar et al., 2025). While NeuroMark-HiFi provided high-resolution characterization of network structure, it did not capture temporal fluctuations. In contrast, NeuroMark-DyFICA (dynamic frequency-informed ICA) extends this framework by incorporating dynamic, windowed ICA and high-pass spatial filtering, enabling detection of fine-scale spatiotemporal variations over time. Thus, NeuroMark-DyFICA preserves the spatial resolution benefits of NeuroMark-HiFi while adding sensitivity to dynamic changes, allowing investigation of transient network reconfigurations that may relate to cognitive flexibility and pathology.

At the same time, conventional dynamic ICA approaches typically emphasize low spatial-frequency or large-scale variations, leaving high-frequency spatial features underexplored. While concerns remain that such fine-scale fluctuations could partly reflect preprocessing artifacts, recent work has provided converging evidence for their neuronal relevance. For example, Iraji and colleagues have shown that transient, fine-grained spatial variations within canonical brain networks are structured, reproducible, and sensitive to individual differences as well as clinical and genetic factors (Iraji et al., 2024; Iraji, Deramus, et al., 2019; Iraji, Fu, et al., 2019). These findings suggest that fluctuations at finer spatial scales are not merely noise but may reflect physiologically meaningful dynamics, underscoring the need for dedicated frameworks to isolate and robustly characterize such high-frequency features. A crucial methodological challenge is therefore to enhance sensitivity to fine spatial scales while maintaining robustness and avoiding potential circularity in data-driven decomposition.

To address these gaps, we propose a novel NeuroMark-DyFICA framework that extends previous spatially dynamic ICA approaches (Iraji et al., 2024) by integrating NeuroMark’s spatial priors with a multistage dynamic pipeline. First, Dynamic NeuroMark ICA is applied within sliding windows to estimate time-resolved networks with cross-subject correspondence, building on prior work while explicitly incorporating high-quality normative spatial priors to improve reproducibility and interpretability. Next, a controlled high-pass spatial filtering step enhances fine-scale, frequency-specific features within these networks. Finally, the latent space of high-frequency dynamics is derived at the group level by applying ICA to the aggregated, filtered maps, yielding refined dynamic components that capture subtle spatial shifts and topographic changes in network organization. This framework improves detection to transient and fine-grained spatiotemporal variations while preserving reproducibility and interpretability across individuals and groups. By combining frequency-informed spatial filtering with spatially constrained dynamic ICA, NeuroMark-DyFICA isolates spatially dynamic patterns that conventional dFNC or sliding-window approaches cannot access.

Unlike prior NeuroMark applications (Du et al., 2020) or NeuroMark-HiFi (Behzadfar et al., 2025), which either emphasized static reproducibility or fine-scale variations in a time-averaged setting, NeuroMark-DyFICA explicitly integrates high-frequency spatial filtering with time-resolved ICA guided by normative priors. This combination creates a novel latent space of high-frequency dynamics that is both reproducible across subjects and uniquely sensitive to transient, subnetwork-level reconfigurations. Importantly, whereas conventional dynamic ICA methods typically capture large-scale shifts, NeuroMark-DyFICA isolates subtle but systematic fine-scale dynamics—changes in spatial topography that unfold over short windows—thus providing access to a dimension of functional brain organization not addressed by existing frameworks.

SZ has been repeatedly associated with subtle disruptions in functional brain networks, many of which may not be captured by traditional temporal connectivity analyses (Iraji, Deramus, et al., 2019). Recent studies demonstrate that spatial dynamics—transient shifts in network topography and fine-scale subspace patterns—can improve detection of SZ-related alterations, revealing differences that are otherwise missed by static or temporal-only approaches (Iraji et al., 2024; Iraji, Fu, et al., 2019). Motivated by this evidence, we applied NeuroMark-DyFICA to SZ data, demonstrating enhanced sensitivity to group differences at previously unstudied fine spatial scales in networks implicated in the disorder. Thus, the key novelty of NeuroMark-DyFICA is that it does not merely combine sliding-window ICA with spatial filtering; instead, it establishes a reproducible latent space of fine-scale, high-frequency dynamics that bridges individual- and group-level analyses. This enables the detection of clinically relevant subnetwork reconfigurations that remain invisible to both static ICA (including NeuroMark) and to temporal-only or low-resolution dynamic methods. In this way, NeuroMark-DyFICA opens a fundamentally new window into the spatial chronnectome of psychiatric disorders.

## METHODS

**Figure 1.**
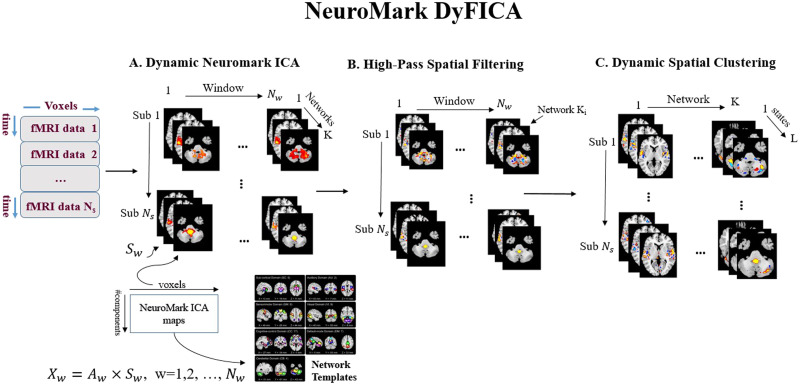
Overview of the proposed NeuroMark-DyFICA framework. The pipeline consists of three stages: (A) Dynamic NeuroMark ICA applies spatially-constrained ICA across sliding windows to estimate time-varying spatial networks (*X*_*w*_ = *A*_*w*_ *S*_*w*_). (B) High-pass spatial filtering enhances fine-scale spatial details in the obtained maps. (C) The latent space of high-frequency dynamics is derived by performing group-level ICA on aggregated and filtered maps, yielding refined dynamic functional patterns and their temporal expression across subjects and windows.

We propose a novel NeuroMark-DyFICA framework designed to enhance sensitivity to dynamic and fine-scale spatiotemporal variations in fMRI data through a multistage spatial filtering pipeline (Figure 1). The workflow consists of three sequential stages. The resulting components represent refined dynamic functional patterns that capture variations within each original network, while the associated mixing weights characterize the temporal expression of these patterns across sliding windows. In contrast to conventional dFNC methods that focus on temporal covariance changes, NeuroMark-DyFICA isolates spatially dynamic fluctuations in network topography, providing a new dimension of spatial chronnectome modeling.

### Stage 1: Dynamic NeuroMark ICA

In the first stage, we implement a dynamic version of NeuroMark ICA, which constrains ICA with the NeuroMark spatial priors across sliding windows to extract time-varying spatial networks. The NeuroMark spatial priors that were derived from large, independent resting-state fMRI datasets comprising thousands of healthy subjects. These priors were established by performing group ICA followed by replication and stability analyses to identify intrinsic connectivity networks that are consistently expressed across populations and imaging conditions.

Within the NeuroMark framework, these priors are incorporated as soft spatial constraints using the Moo-ICAR algorithm, encouraging correspondence with the canonical network patterns while still allowing subject- and window-specific deviations in network topography. This design improves reproducibility and component matching across subjects and windows without imposing fixed spatial patterns.

This process is formalized as:

$$X_{w}=A_{w}\times S_{w},w=1,2,\cdots ,N$$ (1)

where *X*_*w*_ represents the fMRI data within window *w*, *S*_*w*_ is the window-specific spatial maps constrained by the NeuroMark spatial priors, and *A*_*w*_ are the corresponding time courses. This step produces *K* dynamic spatial maps per window, reflecting spatially constrained brain networks that evolve over time (Figure 1A).

While the use of spatial priors enhances stability and cross-subject correspondence, it may bias estimation toward canonical network configurations. To mitigate this effect, subsequent stages of the NeuroMark-DyFICA framework explicitly emphasize fine-scale and high-frequency spatial deviations from the normative spatial priors, thereby enhancing sensitivity to transient and potentially pathological network reconfiguration.

### Stage 2: High-Pass Spatial Filtering

To capture time-resolved fine-scale spatial fluctuations, we next incorporate a 3D high-pass spatial filtering step, which is applied to each of the time-varying network maps *S*_*w*_ obtained from Stage 1. Formally, for each sliding window *w* and network component *k*:

$$S_{w,k}^{HP}=S_{w,k}-\left(S_{w,k}*G_{\sigma }\right)$$ (2)

where *S*_*w*,*k*_ is the spatial map of network component *k* in window *w*, *G*_*σ*_ is a 3D Gaussian smoothing kernel with standard deviation *σ*, * denotes 3D convolution, and $S_{w,k}^{HP}$ is the resulting high-pass filtered spatial map. This step enhances high-frequency spatial details while suppressing low-frequency information, thereby improving sensitivity to subtle, transient spatial fluctuations that may reflect pathological or condition-specific processes (Figure 1B).

We emphasize that high-pass filtering is applied to the ICA-derived network maps, rather than the raw fMRI data. This choice ensures that the filtering operates within the functional topography defined by the NeuroMark spatial priors, enhancing fine-scale spatial deviations within canonical networks. Applying high-pass filtering prior to ICA would remove low-frequency structure from the raw data, potentially altering the global network estimation and reducing correspondence to normative spatial priors. By contrast, filtering after ICA preserves the spatially constrained network patterns while highlighting subtle, high-frequency variations within each network, which are the primary target of our analysis.

High-frequency spatial variations capture fine-grained, transient reconfigurations of functional networks that are relevant to moment-to-moment cognitive processes, perceptual variability, and clinically meaningful alterations in psychiatric and neurological populations (Allen et al., 2014; Iraji, Fu, et al., 2019). In contrast, low-frequency components primarily reflect large-scale network architecture and slow trends, which may obscure subtle deviations in network topography (Leonardi & Van De Ville, 2015). By emphasizing high spatial frequencies, NeuroMark-DyFICA is sensitive to short-lived deviations within canonical networks, enabling detection of subtle abnormalities—such as the imbalance between inactive and hyperengaged states observed in SZ—that would likely be averaged out or smoothed in a low-pass or unfiltered analysis.

### Stage 3: Latent Space of High-Frequency Dynamics

In the final stage, we identify latent subcomponents of the high-frequency spatial dynamics within each spatially constrained network. First, the high-pass filtered spatial maps from all windows and subjects are concatenated into an aggregated matrix of size *V* by (*N*_*w*_ × *N*_*s*_), where *N*_*w*_ is the number of windows, *N*_*s*_ the number of subjects, and *V*is the number of voxels. Next, principal component analysis (PCA) is applied to reduce dimensionality to a target number *L*. Finally, Infomax ICA is performed on the reduced matrix, yielding *L* maximally independent components per spatially constrained network, each represented by a subject-level spatial map *V* by *L* and a corresponding set of mixing weights *L* by (*N*_*w*_ × *N*_*s*_). This procedure enables the extraction of fine-grained spatial subcomponents that are constrained by normative spatial priors but sensitive to transient variability, providing a richer latent representation of network-specific dynamics.

Together, these stages establish a reproducible latent space of frequency-informed spatial dynamics that extends beyond standard dynamic ICA frameworks. The use of independent spatial priors, spatially constrained filtering, and cross-window aggregation supports robustness and minimizes circular dependencies, ensuring that discovered patterns reflect genuine spatiotemporal reconfigurations rather than methodological artifacts.

To assess the reliability of the group-level ICA decomposition in the final stage of the NeuroMark-DyFICA pipeline, we used the ICASSO method (a method for assessing the reliability/stability of ICA estimates) as implemented in the GIFT toolbox. ICASSO evaluates the stability of independent components by comparing results obtained from repeated ICA runs with different initial conditions (Himberg et al., 2004). In our analysis, the group ICA was repeated twice, following the ICASSO implementation in GIFT, and the resulting components were clustered based on spatial similarity. Component stability was quantified using the ICASSO stability index (Iq), which reflects the compactness of each component cluster across runs. Although ICASSO is often applied with a larger number of repetitions, even with two runs, it provides a quantitative measure of reproducibility by explicitly testing consistency across independent ICA initializations. Components with high Iq values indicate robust and reproducible spatial patterns.

### 2D Dynamic Simulation Design

We first implemented a simplified 2D simulation as a proof of concept to clearly demonstrate the methodological advantages of the proposed NeuroMark-DyFICA framework. The 2D setup was deliberately chosen because it provides a transparent and computationally efficient environment, while still allowing systematic control over spatial-frequency content. This makes it possible to isolate and test sensitivity to fine-scale spatial shifts—our primary target—without the additional confounds introduced by hemodynamic convolution, 3D brain anatomy, or physiological noise.

To generate the 2D dataset, we used a controlled fMRI-like dataset on a 2D grid of size *N* × *N* with *N* = 100. The simulation was designed to test whether windowed ICA combined with spatial-frequency filtering enhances sensitivity to subtle spatial shifts that mimic group-level functional differences.

We defined two isotropic Gaussian sources (“blobs”) on the grid, denoted *S*_*A*_(*x*, *y*) and *S*_*B*_(*x*, *y*). Each blob is an isotropic Gaussian with center (*x*_*c*_, *y*_*c*_) and standard deviation *σ* (in pixels),

$$S\left(x,y,x_{c},y_{c},\sigma \right)=\exp\left(-\frac{\left(x-x_{c}\right)+\left(y-y_{c}\right)}{2\sigma^{2}}\right)$$ (3)

Source A was centered at (*x*_*c*_, *y*_*c*_) = (30, 70), and source B at (10, 20) and width *σ* = 5 pixels. Each source was vectorized using the operator *vec*(·) and L_2_-normalized to unit energy:

$$\tilde{S}=\frac{vec\left(S\left(\cdot \right)\right)}{{\left\|vec\left(S\left(\cdot \right)\right)\right\|}_{2}}$$ (4)

To simulate subtle pathology-like differences, we introduced a small horizontal displacement of source A (denoted $S_{A}^{\text{shift}}$):

$$S_{A}^{\text{shift}}\left(x,y\right)=S_{A}\left(x,y-\Delta \right),\Delta =2\;\text{pixels}$$ (5)

This minor horizontal shift (Δ = 2 *pixels*) represents subtle, localized alterations in network topography, analogous to fine-scale spatial variability observed in resting-state fMRI. Such voxel-level topographic fluctuations have been documented in SZ and other clinical populations, where functional network maps vary over time and differ between patients and healthy controls (HCs) (Ma et al., 2014; Pusuluri et al., 2024). By controlling the magnitude of Δ, this simulation allows us to systematically test the sensitivity of NeuroMark-DyFICA to detect small but meaningful spatial shifts while avoiding confounding factors such as hemodynamic convolution, 3D brain anatomy, or physiological noise. This design supports the biological plausibility of our 2D simulation as a proof of concept for evaluating fine-scale network detection.

We then constructed two source matrices by stacking vectorized sources as rows,

$$S_{1}=\left[\begin{array}{c} \overset{´}{{\tilde{S}}_{A}}\\ \overset{´}{{\tilde{S}}_{B}} \end{array}\right],S_{2}=\left[\begin{array}{c} {\tilde{S}}_{A}^{\overset{´}{\text{shift}}}\\ \overset{´}{{\tilde{S}}_{B}} \end{array}\right],S_{1},S_{2}\in R^{2\times P},P=N^{2}$$ (6)

where ′ denotes transpose.

We simulated 200 subject data, divided into two equal groups (Group 1 and Group 2), each with *W* = 40 windows. For each subject *i*, we drew two independent mixing matrices,

$$A_{1}^{\left(i\right)},A_{2}^{\left(i\right)}\approx N\left(0,1\right),A_{j}^{\left(i\right)}\in R^{w\times 2}$$ (7)

that determine how sources vary across windows. To encode group structure, we randomly selected for each subject a fraction of windows generated from the shifted source set *S*_2_:

$$f^{\left(i\right)}\approx \left\{\begin{array}{l} U\left(0.05,0.25\right).\text{Group}\;1.\\ U\left(0.25,0.45\right).\text{Group}\;2 \end{array}\right.$$ (8)

Let $I_{\text{shift}}^{\left(i\right)}\subset \left\{1,\ldots ,W\right\}$ denote the $n_{\text{shift}}^{\left(i\right)}=\left[f^{\left(i\right)}\left(W\right)\right]$ windows using *S*_2_. The observed image at window *w* for subject *i* is:

xwi≈A1iw,:s1,w∉IshiftiA2iw,:s2,w∈Ishifti (9)

To make recovery nontrivial, white Gaussian noise was added to each subject matrix *X*^(*i*)^ ∈ *R*^*W*×*P*^ to achieve a target signal-to-noise ratio (SNR) of 20 dB. For each subject, we computed the empirical signal power *P*_*s*_ = *mean*((*X*^(*i*)^)^2^) and added $N^{\left(i\right)}\approx N\left(0,\sigma_{n}^{2}\right)$ with:

σ=PS10SNRdB10,SNRdB=20 (10)

Yielding noisy data ${\tilde{X}}^{\left(i\right)}=X^{\left(i\right)}+N^{\left(i\right)}$.

Each time window was then high-pass filtered in the Fourier domain to emphasize high-frequency, fine-scale spatial structure. For a 2D fast fourier transform (FFT) with radial frequency *r*, an ideal high-pass filter was applied using a binary annulus mask with cutoff *r*_*c*_, suppressing all Fourier coefficients with *r* ≤ *r*_*c*_. While this sharp frequency separation may introduce minor ringing artifacts, these effects were negligible in our controlled simulation (Equation 11).

$$H\left(u,v\right)=1\left[r>r_{c}\right]$$ (11)

Finally, all filtered maps from every subject and window were concatenated and decomposed with a second ICA to extract consistent group-level patterns across time windows.

To quantify how often each dynamic component dominates the network states across time, we analyzed subject-level mixing weights obtained from the second-level ICA, denoted *A* ∈ *R*^*W*×*S*×*N*^, where *W* is the number of sliding windows, *S* is the number of independent components (states), and *N* is the number of subjects. For each subject *n* and window *w*, we defined:

$$\begin{array}{l} C_{n}^{+}\left(w\right)=\arg\;\max_{S}\;A_{n}\left(w,s\right)\\ C_{n}^{-}\left(w\right)=\arg\;\min_{S}\;A_{n}\left(w,s\right) \end{array}$$ (12)

Here, $C_{n}^{+}\left(w\right)$ and $C_{n}^{-}\left(w\right)$ denote the positively dominant and negatively dominant components respectively. A window was counted to the positive occupancy of component *s* if $s=C_{n}^{+}\left(w\right)$ and *A*_*n*_(*w*, *s*) > 0; it contributed to the negative occupancy if $s=C_{n}^{-}\left(w\right)$ and *A*_*n*_(*w*, *s*) < 0. For each subject, occupancy percentages were computed as:

$$\begin{array}{l} P_{n}^{+}\left(s\right)=\frac{O_{n}^{\left(+\right)}\left(s\right)}{W}\times 100\\ P_{n}^{-}\left(s\right)=\frac{O_{n}^{\left(-\right)}\left(s\right)}{W}\times 100 \end{array}$$ (13)

where $O_{n}^{\left(+\right)}\left(s\right)$ and $O_{n}^{\left(-\right)}\left(s\right)$ are the counts of windows meeting the respective criteria.

Occupancy percentages were subsequently split by group for statistical comparison. For each component *s*, group differences in $P_{n}^{\left(+\right)}\left(s\right)$ and $P_{n}^{\left(-\right)}\left(s\right)$ were assessed using two-sample *t* tests, and effect sizes were summarized as signed −*log*_10_(*p*) values:

$${\text{stat}}_{s}\left(\pm \right)=-{\text{log}}_{10}\left(P_{s}\left(\pm \right)\right)\cdot \text{sign}\left(t_{s}\left(\pm \right)\right)$$ (14)

where *p*_*s*_ and *t*_*s*_ are the *p* value and *t* statistic for the corresponding comparison. This formulation preserves sign information and avoids additional normalization or magnitude operations, aligning with our signed-weight occupancy definition.

This continuous pipeline demonstrates, in a simple setting, how the proposed NeuroMark-DyFICA method—combining windowed ICA and spatial filtering—can improve detection of subtle group differences driven by fine-scale spatial shifts in network topography. It allows quantification of how frequently each dynamic state dominates across subjects and windows, separately for positive and negative excursions of the component weights, providing a fine-grained metric of spatial occupancy in high-frequency brain dynamics.

### Analysis of fMRI Data

We next analyzed resting-state fMRI data from 131 SZ patients and 141 HCs obtained from the Phase III dataset of the Functional Biomedical Informatics Research Network (Potkin & Ford, 2009). The acquisition and preprocessing details have been described in detail previously; here we briefly summarize them for clarity (Damaraju et al., 2014). fMRI volumes were collected across seven sites using 3T MRI scanners with an echo planar imaging (EPI) sequence (field of view: 220 × 220 mm, matrix: 64 × 64, repetition time = 2 s, echo time = 30 ms, flip angle = 77°, 162 volumes, 32 axial slices of 4 mm thickness with 1 mm gap). Subjects were instructed to keep their eyes closed throughout the scanning session. Preprocessing was carried out using SPM12 (https://www.fil.ion.ucl.ac.uk/spm/) and included motion correction, slice-timing correction, and normalization to the standard Montreal Neurological Institute (MNI) space using an EPI template. To reduce intersite variability in spatial smoothness, all data were spatially smoothed to 6 mm full width at half maximum (FWHM) using Analysis of Functional NeuroImages (AFNI’s) Blur to FWHM algorithm, which provides conservative denoising and “smoothness equivalence” across multisite datasets. We note that this preprocessing follows the pipeline described in prior work (Damaraju et al., 2014). The choice of 6 mm FWHM represents a common compromise between noise reduction and preservation of fine-grained spatial detail.

For applying dynamic NeuroMark ICA, the preprocessed fMRI time series for each subject were segmented into overlapping temporal windows to capture time-varying connectivity patterns. Each window comprised 60 consecutive time points (approximately 2 min, given TR = 2 s), and consecutive windows were shifted by 2 time points. This stride size was chosen as a balance between maximizing the number of windows (and thus temporal sensitivity) and computational feasibility: while a stride of 1-time point would yield slightly finer sampling, it would also produce nearly double the number of windows (98 instead of 49), leading to substantially higher memory demands without appreciable gain in information content due to the strong overlap between adjacent windows. With 157 total time points, this configuration produced 49 sliding windows per subject, which were then used as input for the spatially constrained ICA decomposition, enabling estimation of time-resolved spatial networks that evolve across windows while maintaining correspondence across subjects. We note that the choice of window length can influence the sensitivity to temporal dynamics: shorter windows may capture more rapid fluctuations but increase noise and reduce stability of ICA estimates, whereas longer windows provide more stable estimates but may smooth over fast transitions. Our selection of 60 TR (approximately 2 min) reflects a balance between temporal resolution and estimation reliability, consistent with prior dynamic connectivity studies (Allen et al., 2014).

After windowing, NeuroMark ICA was applied to each windowed segment of every subject using the spatially constrained ICA implementation in GIFT software at https://trendscenter.org/software/gift (Adali, 2012; Iraji et al., 2021). This framework incorporates spatial priors derived from large independent datasets, enabling automated estimation and labeling of subject-specific connectivity features. ICA decomposition was performed with the Moo-ICAR algorithm, and template matching used the NeuroMark_fmri_1.0 template (Du et al., 2020) (available at https://trendscenter.org/data and in the GIFT software) to identify intrinsic connectivity networks. For each window, 53 (*k*) networks were extracted, which served as spatially constrained networks for the subsequent stages of the NeuroMark-DyFICA analysis. This approach ensures that the resulting spatial maps reflect time-resolved network patterns while maintaining correspondence across subjects.

Next, we applied a 3D high-pass spatial filter in the Fourier domain to each ICA-derived network maps from Stage 1. Specifically, each 3D spatial map was Fourier-transformed, and multiplied by a binary ideal high-pass filter, retaining only Fourier components with radial frequency *r* > *r*_*c*_, where *r*_*c*_ = 7 voxel-frequency units (0.031 mm^−1^). This sharply removes low-frequency spatial content while preserving high-frequency details that reflect fine-scale topographic fluctuations. Following masking, the filtered maps were transformed back to the spatial domain. The cutoff value was selected based on prior optimization (Behzadfar et al., 2025), where different thresholds were evaluated and *r*_*c*_ = 7 was found to effectively preserve fine spatial details while suppressing global low-frequency structure. Minor ringing artifacts due to the ideal filter were observed in simulations but were negligible. The retained frequency band reflects the combined effect of the original 6 mm FWHM Gaussian smoothing applied during preprocessing and this high-pass filter, ensuring that fine-scale spatial features were emphasized while larger-scale variations were minimized.

Next, we refined the dynamic spatial patterns within each spatially constrained network using a group-level ICA decomposition implemented in GIFT (MATLAB). To prepare the data, the dynamic windowed maps from all subjects were concatenated across time, and PCA was applied to reduce dimensionality while retaining maximum variance. These reduced components were then entered into a group ICA, performed with the Infomax algorithm as implemented in GIFT (Calhoun et al., 2001). To obtain subject-specific representations, a back-reconstruction procedure was used, which reverses the PCA steps and projects the group-level components back into individual space, ensuring accurate estimation of subject-level dynamic maps (Allen et al., 2011; Erhardt et al., 2011). For each spatially constrained network, the model order was fixed at five refined dynamic subcomponents (*L = 5*), determined using the elbow method applied to the PCA scree plot. This approach balances variance explained with dimensionality reduction and avoids overfitting. Selecting five subcomponents is consistent with prior studies demonstrating that a small number of components is sufficient to capture the majority of meaningful spatial variability within functional networks while maintaining interpretability (Calhoun et al., 2009). These subcomponents were carried forward to subsequent analyses. To evaluate group-level differences, we employed two complementary measures introduced in Soleimani et al. (2025): dynamic state density and dynamic convergence/divergence.

### Dynamic State Density

This measure characterizes the distribution of “surviving” states across groups based on time-varying connectivity patterns. In our analysis, the number of states was set to *L* = 5, with a total of *N*_*w*_ = 49 windows per subject. For each window *w* and state *l*, a binary indicator *I*_*w*,*l*_ was defined by applying a threshold (*thr* = 1) to the absolute value of the component weights, such that *I*_*w*,*l*_ = 1 if |*I*_*w*,*l*_| > *thr* and 0 otherwise. This threshold was chosen based on the normalized component weight distributions across all subjects and windows, ensuring that only sufficiently strong and meaningful contributions of a component to the network state were considered. Sensitivity analyses in our preliminary experiments showed that small variations around this threshold did not substantially affect the identification of dominant states, indicating robustness of this choice. The number of strong states at window *w* was then computed as:

$$S_{w}=\sum_{l=1}^{L}I_{w,l}$$ (15)

A histogram of *S*_*w*_ (bins 0 to *L*) was generated for each subject, normalized by the total number of windows *W*, and averaged across subjects within each group:

$$P\left(S=k\right)=\frac{1}{n}\sum_{i=1}^{n}P_{i}\left(S=k\right),k\in \left\{0,1,\cdots ,L\right\}$$ (16)

where *n* is the number of subjects in the group, *L* is the number of states/components, and *P*_*i*_(*S* = *k*) is the normalized histogram for subject *i*. To compare patient groups with HCs, we employed a general linear model for each bin, with diagnosis (SZ vs. HC) as the primary predictor and age, sex, and scanning site included as covariates to control for potential confounding effects. Statistical inference was based on the significance of the diagnosis term, yielding group-level effect estimates and *p* values. This analysis captures differences in the distribution of dynamic states while accounting for demographic and site-related variability, thereby highlighting transient connectivity patterns that discriminate patients from controls.

### Dynamic Convergence/Divergence

This measure evaluates the stability and variability of state contributions over time by examining how strongly or weakly states co-activate. For each individual *i*, we considered the mixing matrix *x*_*i*_ ∈ *R*^*W*×*L*^, representing the activation strengths of *L* = 5 states across *W* windows. Each window at time *t* was represented by a feature vector *x*_*i*_(*t*) ∈ *R*^*L*^.

To quantify similarity between states, the Euclidean distance between any two states *x*_*i*_(*t*) and *x*_*j*_(*t*) at each time point was computed as:

$$d_{ij}\left(t\right)=|\left|x_{i}\left(t\right)-x_{j}\left(t\right)\right||$$ (17)

States were classified as converged if *d*_*ij*_(*t*) ≤ *ε* and diverged if *d*_*ij*_(*t*) ≥ *θ*, where thresholds *ε* and *θ* were empirically determined from the distribution of pairwise differences across subjects and time points. Specifically, we computed the distribution of all pairwise state distances and selected *ε* as the 10th percentile (capturing close or highly similar states) and *θ* as the 90th percentile (capturing strongly separated states). This percentile-based choice is data-adaptive and avoids arbitrary thresholds; by using symmetric tails (10th and 90th percentiles), it captures meaningful deviations at both extremes while excluding central noise. We also tested modest shifts in these cutoffs (e.g., 5th/95th, 15th/85th) and found that the main results—namely, which convergence/divergence pairs were significant—remained qualitatively consistent, supporting the robustness of our threshold selection. This approach provides a principled way to define convergence and divergence events, enabling reliable tracking of whether brain connectivity states align (converge) or separate substantially (diverge), reflecting adaptive or maladaptive reconfigurations. By combining state density and convergence/divergence analyses, we capture both the frequency distribution of transient states and the temporal flexibility of state relationships, providing a more comprehensive characterization of group differences in dynamic connectivity.

## RESULTS

### Simulation

**Figure 2.**
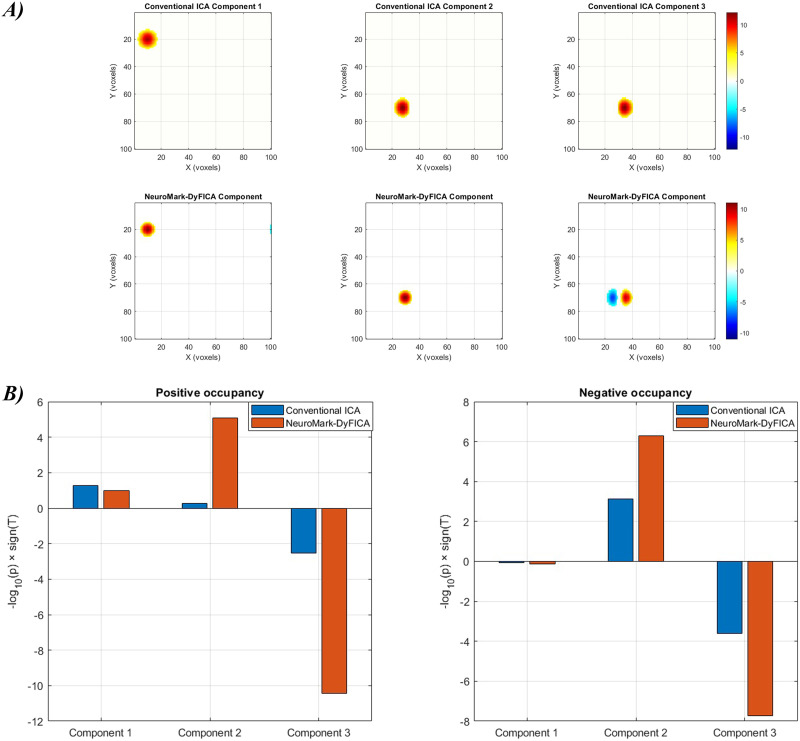
(A) Spatial maps of the three independent components. The first row shows results from the conventional ICA method, while the second row shows results from the NeuroMark-DyFICA method. Component 1 corresponds to Source B, which has no spatial shift. Component 2 corresponds to Source A with a horizontal spatial shift. Component 3 represents a bimodal component combining both original and shifted versions of Source A. Color bar shows voxel-wise component weights. (B) Statistical comparison of group difference detection between NeuroMark-DyFICA (red) and conventional (blue) ICA methods. Bar plots show test statistics (−*log*_10_(*p*) × *sign*(*T*)) for positive and negative occupancy measures, demonstrating NeuroMark-DyFICA’s enhanced ability to detect subtle spatial shifts and group differences in Components 2 and 3.

We compared the proposed NeuroMark-DyFICA framework with conventional group ICA in their ability to detect group differences arising from subtle spatial shifts in simulated fMRI data. The number of components for both methods was set to three, corresponding to the two original Gaussian sources (A and B) and the horizontally shifted version of source A (A’), which was introduced to mimic subtle group-level spatial differences. Component-specific positive and negative occupancy measures were then computed for each method. For each component, we computed a test statistic combining the significance and direction of group differences, expressed as −*log*_10_(*p*) × *sign* (*T*), where *p* is derived from a two-sample *t* test and *T* is the corresponding *T* value (Figure 2). All statistical tests were corrected for multiple comparisons using false discovery rate (FDR) correction to ensure robustness of the reported effects.

Component 1 corresponds to Source B, which contained no spatial shift between groups. As expected, neither NeuroMark-DyFICA nor conventional ICA revealed significant group differences for either positive or negative occupancy measures.

This result confirms that both methods maintain specificity and accuracy in detecting true spatial differences when no spatial shifts are present.

Component 2 represents Source A, which incorporated a subtle horizontal shift between groups to mimic a pathology-like effect. Here, NeuroMark-DyFICA demonstrated greater detection power, identifying significant group differences for both positive and negative occupancy measures. In contrast, conventional ICA only identified a significant difference for negative occupancy, and this effect was substantially smaller in magnitude than that observed with NeuroMark-DyFICA. This indicates that while conventional ICA partially captured the spatial shift, it failed to detect the full extent of the group difference, particularly in the positive direction.

Component 3 captures a bimodal mixture of the original Source A and its shifted version. For this complex spatial pattern, NeuroMark-DyFICA exhibited markedly stronger significance for both positive and negative occupancy compared with conventional ICA. The test statistics for NeuroMark-DyFICA were substantially larger in magnitude, reflecting its enhanced ability to resolve overlapping spatial configurations and extract meaningful group-level differences from mixed spatial representations.

Across all components, these results demonstrate that NeuroMark-DyFICA consistently outperformed conventional group ICA in detecting subtle spatial differences between groups. The framework’s spatial-frequency filtering step improved the ability to resolve fine-scale spatial variations without introducing spurious effects, as evidenced by the absence of false positives in Component 1. Importantly, NeuroMark-DyFICA’s superior performance was most pronounced in components containing spatial shifts (Components 2 and 3), where it captured both positive and negative aspects of group differences that conventional ICA either partially missed or detected with substantially reduced sensitivity. These findings validate the NeuroMark-DyFICA approach as a more powerful method for identifying subtle spatial variations in dynamic functional imaging data.

### Analysis of fMRI data

**Figure 3.**
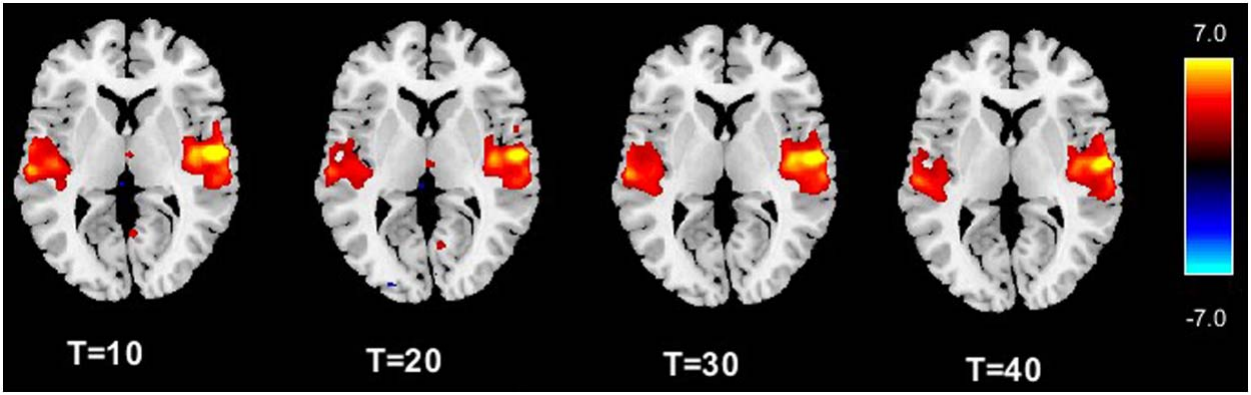
Subject-level dynamic spatial maps for NeuroMark Network 6 (Auditory). Maps are shown for Subject 272 across four sliding windows (10, 20, 30, and 40), illustrating temporal variability in spatial patterns within the same network. A display threshold of 3 was applied (this threshold was selected to highlight the most salient spatial patterns while suppressing background noise), and the color bar represents the *Z* score.

To illustrate the initial outputs of the NeuroMark-DyFICA framework, we first examined subject-level spatial maps derived from the Dynamic NeuroMark ICA stage. Figure 3 shows the extracted maps for a subject (Subject 272) in Network 6 (Auditory) across four different sliding windows (Windows 10, 20, 30, and 40). The results demonstrate that while the same network structure is preserved across windows, subtle fluctuations in spatial topography emerge over time, highlighting the ability of the dynamic approach to capture temporal variability within a single subject.

**Figure 4.**
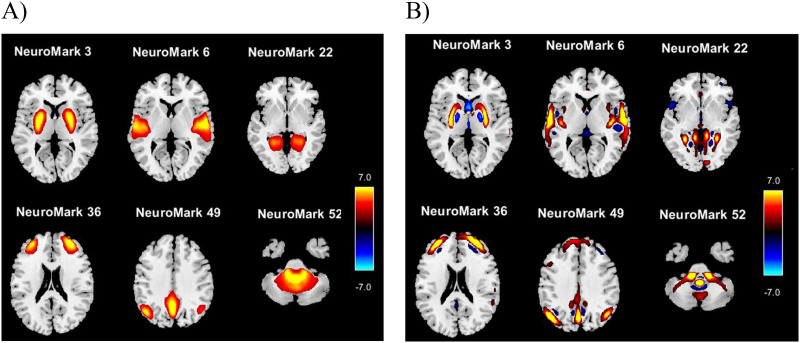
NeuroMark-DyFICA components for six networks before and after high-pass spatial filtering. Group-level mean spatial maps within a single window are shown before and after high-pass spatial filtering. (A) Mean spatial maps across all subjects for six selected NeuroMark networks (3: thalamus; 6: auditory; 22: visual/fusiform; 36: middle frontal; 49: DMN; 52: cerebellum) derived from Dynamic NeuroMark ICA. (B) Corresponding mean spatial maps within the same window after high-pass spatial filtering, demonstrating improved fine-scale spatial resolution. A display threshold of 3 was applied for the maps before filtering, while after filtering a threshold of 2 was used for network 3 and 1 for the remaining networks. The color bar represents the *Z* score.

We next assessed group-level spatial patterns to evaluate the impact of the high-pass spatial filtering stage. Figure 4 presents the mean spatial maps of all subjects within a single window for six representative networks: Network 3 (thalamus), Network 6 (auditory), Network 22 (visual/fusiform), Network 36 (middle frontal), Network 49 (default mode network; DMN), and Network 52 (cerebellum). Figure 4A shows the mean spatial maps obtained directly after Dynamic NeuroMark ICA within the single window; and Figure 4B displays the corresponding maps within the same window after applying the high-pass spatial filtering step. The *Z* scores shown in Figure 4 reflect voxel-wise deviations relative to the component mean, where positive values indicate additive contributions and negative values indicate subtractive contributions to the spatial pattern. After high-pass spatial filtering, negative *Z* scores can be interpreted as voxels contributing with opposite sign weighting at the retained spatial frequencies, reflecting fine-scale spatial contrasts rather than true physiological deactivation. As expected, the filtering procedure accentuates fine-scale spatial details by suppressing low-frequency structure, resulting in sharper, more localized spatial features. While Figure 4 focuses on a single representative window to highlight the spatial effects of filtering at the group level, temporal variability across multiple windows is explicitly illustrated at the subject level in Figure 3, demonstrating NeuroMark-DyFICA’s ability to capture dynamic spatial reconfigurations over time. These results demonstrate how the second stage of the NeuroMark-DyFICA pipeline improves the detection of subtle, transient spatial fluctuations while maintaining correspondence across subjects.

**Figure 5.**
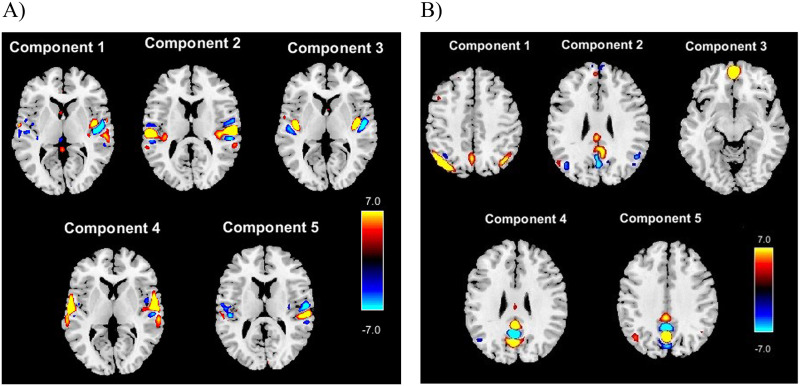
NeuroMark-DyFICA components for the auditory network (NeuroMark 6) and the DMN (NeuroMark 49). Example results from the group-level ICA decomposition of these two networks. (A) Five independent components identified within the auditory network, illustrating refined subnetworks of the auditory system. (B) Five independent components identified within the DMN, illustrating refined subnetworks of the default mode system. A display threshold of 3 was applied, and the color bar represents the *Z* score.

As an illustrative example of the final stage of the NeuroMark-DyFICA pipeline, we display the group-level ICA decomposition results for two NeuroMark networks—the Auditory network (NeuroMark 6) and the DMN (NeuroMark 49). In both cases, five components were extracted, and Figure 5 presents their spatial maps. To evaluate reproducibility, the ICA decomposition was repeated 10 times with different random initializations, and component stability was assessed using the ICASSO framework. All extracted components showed high stability, with Iq exceeding 0.9 for both networks. These results demonstrate that the identified fine-grained subdivisions within spatially constrained networks are robust to ICA initialization and reproducible across runs, supporting the reliability of the NeuroMark-DyFICA framework. These results highlight how NeuroMark-DyFICA captures fine-grained subdivisions within spatially constrained networks, demonstrating the framework’s ability to resolve distinct dynamic patterns that may not be evident in the original normative spatial priors.

Before running the full NeuroMark-DyFICA pipeline, we selected six NeuroMark components based on prior evidence of their alteration in SZ. Studies have repeatedly shown thalamocortical dysconnectivity (thalamic/subcortical) in SZ (Anticevic et al., 2014; Giraldo-Chica & Woodward, 2017; Woodward et al., 2012), altered visual/fusiform cortex activity and connectivity (He et al., 2024; Iwabuchi & Palaniyappan, 2017; Maher et al., 2019; Silverstein & Keane, 2011), DMN disruptions (Sasabayashi et al., 2023; Whitfield-Gabrieli & Ford, 2012), auditory and temporal abnormalities including hallucinations associated cortical regions (Alhazmi, 2025; Williams et al., 2025; Zhang et al., 2025), frontal cortex dysfunction (executive control, working memory) (Jiang et al., 2019), and cerebellar involvement in resting-state connectivity and regulation of cognition in SZ (Forlim et al., 2024; Kim et al., 2020; Park et al., 2021).

**Figure 6.**
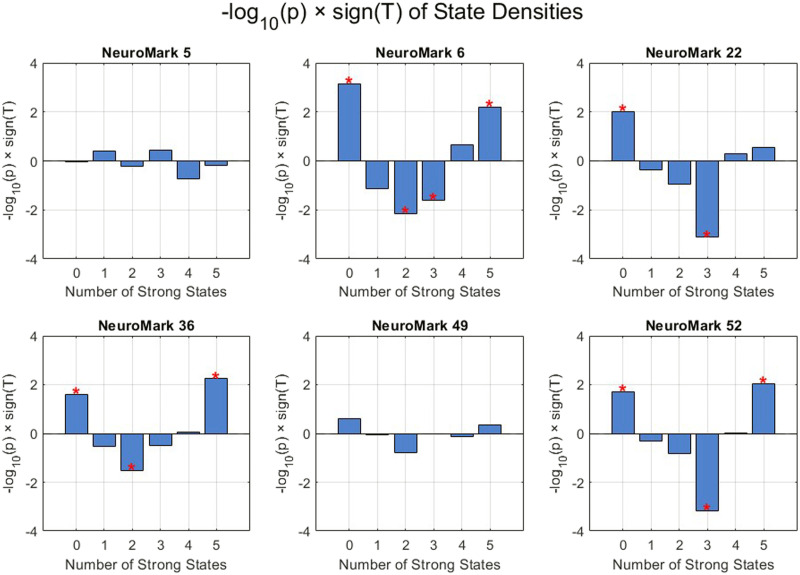
Results of the dynamic state density analysis for the six selected NeuroMark networks: thalamus (NeuroMark 3), auditory (NeuroMark 6), visual/fusiform (NeuroMark 22), middle frontal (NeuroMark 36), DMN (NeuroMark 49), and cerebellum (NeuroMark 52). Each bar plot shows the signed statistical comparison between SZ patients and HCs, computed as −log_10_(p) × sign(T) for the number of strong states (*x*-axis). Positive values indicate higher prevalence in SZ, while negative values indicate higher prevalence in HCs. Asterisks (*) mark bins where group differences are statistically significant at *p* < 0.05.

Accordingly, we selected these six networks: subcortical/thalamic (Network 3), auditory/temporal (Network 6), visual/fusiform (Network 22), middle frontal (Network 36), DMN (Network 49), and cerebellar (Network 52). All subsequent dynamic analyses were restricted to this literature-driven predefined set. We first assessed group-level differences using the dynamic state density analysis. Figure 6 summarizes the results for the six representative NeuroMark networks: (Network 3, 6, 22, 36, 49, and 52). For each network, the bar plots display the signed test statistic (−log_10_(*p*) × *sign*(*T*)) across the possible numbers of strong states. Positive values correspond to higher prevalence of strong-state configurations in SZ patients, whereas negative values indicate greater prevalence in HCs. Several networks showed significant group differences (marked by *), highlighting altered distributions of dynamic states in SZs compared with HCs.

**Figure 7.**
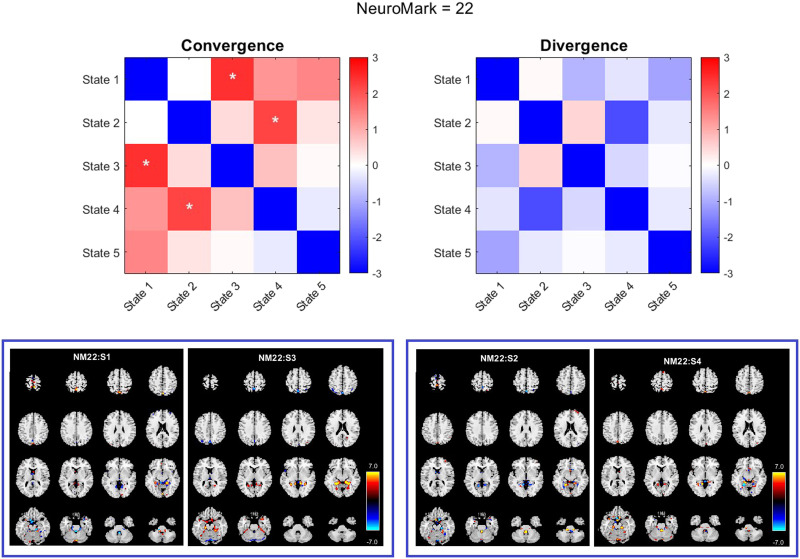
Convergence/divergence analysis results for the visual/fusiform network (NeuroMark 22). The first row shows group-level convergence (left) and divergence (right) matrices, with warm colors (positive values) indicating stronger effects in SZ and cool colors (negative values) indicating stronger effects in HCs. Asterisks (*) mark state pairs with significant group differences after FDR correction (*p* < 0.05). The second row displays the spatial maps of dynamic components associated with the significant state pairs: NeuroMark 22, State 1 (NM22:S1) versus State 3 (NM22:S3), and NeuroMark 22, State 2 (NM22:S2) versus State 4 (NM22:S4). These maps provide anatomical context for the observed abnormalities. A display threshold of 3 was applied, and the color bar represents the *Z* score.

**Figure 8.**
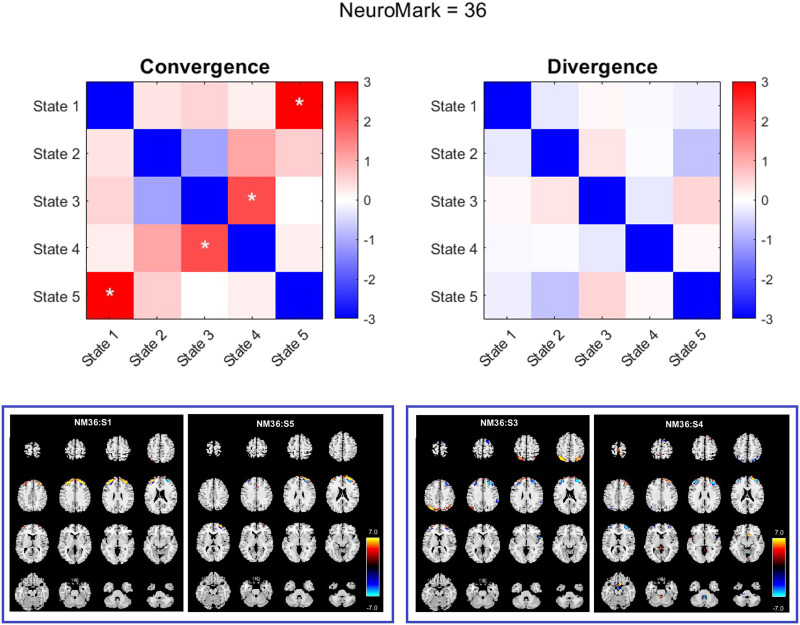
Convergence/divergence analysis results for the middle frontal network (NeuroMark 36). The first row shows group-level convergence (left) and divergence (right) matrices, with warm colors (positive values) indicating stronger effects in SZ and cool colors (negative values) indicating stronger effects in HCs. Asterisks (*) mark state pairs with significant group differences after FDR correction (*p* < 0.05). The second row displays the spatial maps of dynamic components corresponding to the significant state pairs: NeuroMark 36, State 1 (NM36:S1) versus State 5 (NM36:S5), and NeuroMark 36, State 2 (NM36:S3) versus State 4 (NM36:S4). These maps provide anatomical context for the observed abnormalities. A display threshold of 3 was applied, and the color bar represents the *Z* score.

**Figure 9.**
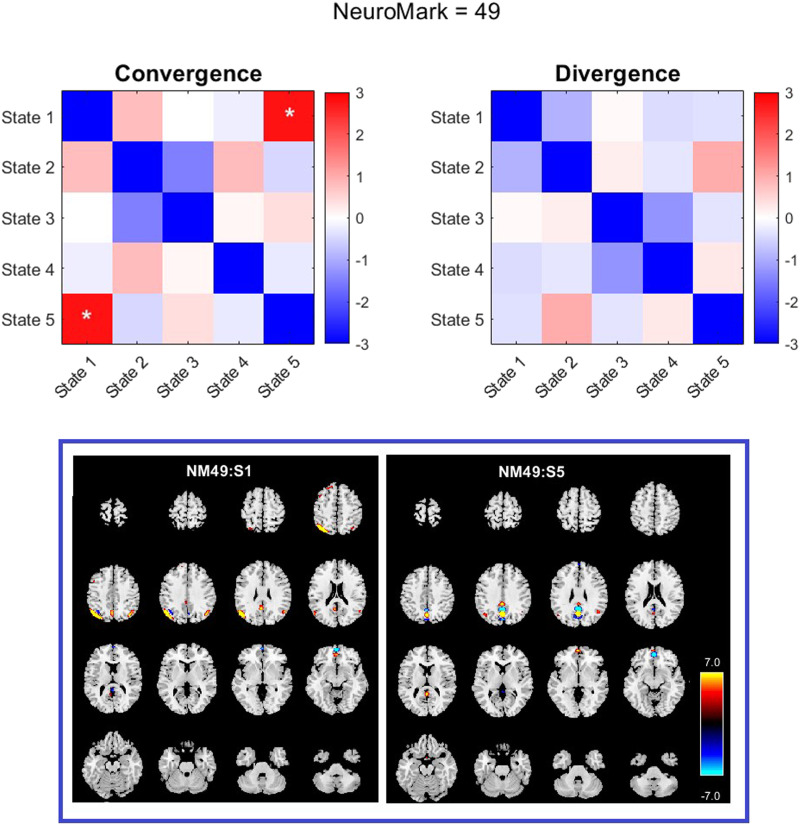
Convergence/divergence analysis results for the DMN (NeuroMark 49). The first row shows group-level convergence (left) and divergence (right) matrices, with warm colors (positive values) indicating stronger effects in SZ and cool colors (negative values) indicating stronger effects in HCs. Asterisks (*) mark state pairs with significant group differences after FDR correction (*p* < 0.05). The second row displays the spatial maps of dynamic components corresponding to the significant state pairs: NeuroMark 49, State 1 (NM49:S1) versus State 5 (NM49:S5). These maps provide anatomical context for the observed abnormalities. A display threshold of 3 was applied, and the color bar represents the *Z* score.

We next applied the convergence/divergence analysis to the same set of networks. Across all analyzed systems, significant group differences emerged most consistently in the visual/fusiform (NeuroMark 22), middle frontal (NeuroMark 36), and DMN (NeuroMark 49) networks. Results for these networks are presented in Figures 7–9. In each figure, the first row shows the convergence (left panel) and divergence (right panel) matrices, summarizing group-level comparisons across all dynamic state pairs. Warm colors indicate stronger effects in SZs, while cool colors indicate stronger effects in HCs. Asterisks mark state pairs with statistically significant differences after FDR correction (*p* < 0.05). These findings reveal that specific pairs of states exhibit altered synchronization (convergence) in SZs relative to HCs, suggesting abnormal temporal reconfigurations of connectivity states as a characteristic feature of the disorder. To further investigate these effects, we examined which network pairs showed the highest differences. Accordingly, in the second row of Figures 7–9, we display the spatial maps of dynamic components corresponding to the state pairs with significant group differences, allowing us to identify the networks most strongly contributing to altered temporal coordination. For example, in NeuroMark 22, significant effects were observed for state pairs (1,3) and (2,4), pointing to disrupted coordination between canonical visual/fusiform states and their dynamic variants. Similarly, NeuroMark 36 and 49 show altered convergence between selected state pairs, providing anatomical context for the functional patterns underlying the observed abnormalities. Together, these results highlight that beyond differences in state prevalence, SZ is also characterized by disrupted coordination between states within key visual, frontal, and default mode systems.

## DISCUSSION

In this study, we introduced NeuroMark-DyFICA, a novel dynamic ICA framework designed to improve the detection of fine-scale spatiotemporal variations in fMRI data. The method integrates spatially constrained dynamic ICA, high-pass spatial filtering, and group-level decomposition in a multistage pipeline, enabling the extraction of refined dynamic functional components. The ability to capture spatial dynamics—moment-to-moment changes in the spatial expression, boundary, and topography of networks—is increasingly recognized as critical for understanding flexible brain function and its breakdown in disorders such as SZ. Prior work has shown that these transient spatial variations can reveal abnormalities not detectable with static analyses. For example, Iraji, Deramus, et al. (2019) demonstrated that patients with SZ exhibit short-lived decreases in voxel-wise network coupling in visual and auditory networks, which static analyses overlook. Similarly, recent studies of spatiotemporal dynamics in functional connectivity found increased spatial variation in perceptual and attentional systems in SZ, pointing to spatial instability in those networks (Hou et al., 2023). Moreover, independent vector analysis has revealed greater fluctuations in spatial concordance among frontoparietal, cerebellar, and temporal regions in SZ compared with controls (Ma et al., 2014), and additional studies have observed increased spatial variability or altered spatial–temporal concordance in SZ (Gopal et al., 2016; Zhu et al., 2018).

However, conventional ICA and connectivity analyses typically average over time or apply spatial smoothing, which can blur or suppress these transient but meaningful fluctuations. Evidence shows that spatial maps change over time: for instance, sliding-window ICA reveals temporal fluctuations in the spatial layout of the DMN (Kiviniemi et al., 2011), and hierarchical domain models demonstrate spatial fluidity within and between functional domains in SZ (Iraji, Fu, et al., 2019). In addition, spatial smoothing has been shown to reduce fine-scale detail, further limiting the ability to detect high-frequency spatial features (Chen & Calhoun, 2018). By contrast, NeuroMark-DyFICA simultaneously leverages prior neurobiological constraints while preserving high-frequency spatial information, enabling the detection of subtle but clinically relevant alterations in brain network dynamics. At the same time, it is important to recognize that the use of NeuroMark introduces spatial priors derived from large independent datasets. These priors provide strong anatomical correspondence and improve reproducibility across subjects and windows, but they may also bias estimation toward canonical network configurations. NeuroMark-DyFICA addresses this trade-off by applying subsequent high-pass spatial filtering and group-level decomposition to emphasize deviations from the normative spatial priors, thereby enhancing sensitivity to dynamic and fine-scale spatial variations while retaining biologically meaningful constraints. This balance between stability and flexibility is particularly important in clinical populations such as SZ, where abnormalities may manifest as subtle deviations from otherwise preserved network architectures.

To validate the framework in a controlled setting, we simulated an fMRI-like dataset with isotropic Gaussian sources on a 2D grid. By introducing subtle spatial shifts between groups, the simulation tested whether NeuroMark-DyFICA could capture small but systematic differences in spatial organization. The results confirmed that combining windowed ICA with high-pass spatial filtering markedly improved the detection of these fine-scale shifts, while conventional ICA failed to detect them reliably. This proof of concept demonstrates the capacity of NeuroMark-DyFICA to recover subtle spatial alterations that mimic pathology-related effects, supporting its application to real-world clinical datasets.

Building on these simulation results, and to further demonstrate the utility of the approach, we next applied NeuroMark-DyFICA to a large resting-state fMRI dataset from SZ patients and HCs. Our analysis began with an exploratory screening of all 53 spatially constrained networks using two independent approaches, dynamic state density analysis and convergence/divergence analysis, to identify candidate systems showing robust group differences. Based on prior evidence implicating specific functional systems in SZ, together with their consistent spatial clarity in our dataset, we selected six representative networks spanning key domains (thalamus, auditory, visual/fusiform, middle frontal, default mode, and cerebellar) for detailed investigation. The subsequent results highlight both network-specific and cross-system alterations in dynamic brain organization associated with SZ.

The dynamic state density analysis revealed network-specific imbalances in the distribution of strong states between SZ patients and HCs. Importantly, the sign of the statistic −log_10_(*p*) × *sign*(*T*) indicates the direction of the effect, with positive values reflecting higher prevalence in SZ and negative values reflecting higher prevalence in HCs. In the auditory network (Network 6), SZ patients showed both increased periods with no strong states (#states = 0) and stronger expression of complex states (#states = 5), suggesting a dual association with SZ: greater inactivity alternating with episodes of hyper-engaged auditory dynamics. By contrast, States 2 and 3 were more prevalent in HCs, consistent with more balanced intermediate auditory states in the control group. The visual/fusiform network (Network 22) showed a similar pattern. SZ patients again exhibited more time in inactive periods (#states = 0), while HCs had greater prevalence of transient higher-order states (#states = 3). This imbalance indicates that in SZ, the visual/fusiform system is more often disengaged, whereas controls display richer dynamic transitions. For the middle frontal network (Network 36), SZ patients demonstrated increased time in both inactive states (#states = 0) and hyper-engaged states (#states = 5), while HCs showed greater prevalence of intermediate frontal states (#states = 2). This pattern points to instability in frontal dynamics in SZ, with oscillations between quiescence and overactivation rather than stable engagement. Finally, in the cerebellar network (Network 52), SZ patients again spent more time in inactive states (#states = 0) and high-amplitude states (#states = 5), while HCs showed more balanced representation of intermediate states such as State 3. These findings suggest that cerebellar contributions in SZ are either underexpressed or overexpressed, reflecting impaired regulation of dynamic cerebellar involvement.

Taken together, these results indicate that SZ is marked not by uniform disruption across networks, but by a recurring imbalance between inactivity (#states = 0) and hyper-engaged dynamics (e.g., #states = 5) across multiple systems (auditory, visual, frontal, and cerebellar). In contrast, HCs maintain a richer distribution of intermediate states (e.g., #states = 2 #states = 3), which may support more flexible and adaptive network behavior. This imbalance provides a mechanistic explanation for the reduced functional adaptability often reported in SZ and highlights the ability of NeuroMark-DyFICA to uncover fine-grained spatiotemporal alterations. These findings are consistent with prior dynamic functional connectivity studies reporting reduced flexibility and abnormal network switching in SZ (Allen et al., 2014; Damaraju et al., 2014; Preti et al., 2017). In particular, our observation of oscillations between inactivity and hyper-engaged states across auditory, visual, frontal, and cerebellar networks aligns with reports of network instability and disrupted transitions in SZ (Iraji et al., 2020; Kottaram et al., 2019; Ramirez-Mahaluf et al., 2023; Rashid et al., 2014; Yang et al., 2022; You et al., 2022).

The convergence analysis revealed significant group differences in how dynamic states aligned over time, while no significant effects were observed for divergence. Specifically, altered convergence was observed in the visual/fusiform network (Network 22; State Pairs 1–3 and 2–4), the middle frontal network (Network 36; Pairs 1–5 and 3–4), and the DMN (Network 49; Pair 1–5). The spatial maps of these components (Figure 7) provide insight into the anatomical basis of these effects. In Network 22, group differences between component pairs (1–3 and 2–4) suggest that SZ patients show abnormal coordination between canonical occipital–fusiform states and their dynamic variants. Given that the fusiform gyrus is crucial for higher-order visual integration, including face and object recognition, these disrupted convergence patterns may reflect impaired integration of visual input into meaningful percepts. This interpretation is consistent with prior reports of fusiform and occipital dysfunction in SZ, including abnormal face processing and visual recognition deficits (Dong et al., 2018; Quintana et al., 2003; Rashid et al., 2014; Sehatpour et al., 2010). In Network 36, altered convergence between frontal state pairs (1–5 and 3–4) indicates abnormal coordination between baseline and more engaged frontal dynamics, aligning with evidence of executive control and prefrontal instability in SZ (Baker et al., 2014; Damaraju et al., 2014; Iraji et al., 2020; You et al., 2022). Finally, in the DMN (Network 49), differences in convergence between States 1 and 5 suggest disrupted regulation between core DMN activity and its high-expression variants. This finding is consistent with the well-documented DMN dysregulation in SZ, where patients show abnormal switching between rest-related and task-negative states (Mingoia et al., 2012; Rashid et al., 2014; Rotarska-Jagiela et al., 2010; Whitfield-Gabrieli & Ford, 2012). Together, these findings extend prior dynamic functional connectivity work by demonstrating that SZ patients not only differ in the frequency distribution of dynamic states (as shown in the state density analysis), but also in the coordination and convergence relationships among states within key systems. This dual disruption—spanning visual/fusiform, frontal, and DMN—supports the broader view of SZ as a disorder of impaired network flexibility and adaptive integration (Calhoun et al., 2014; Damaraju et al., 2014).

Taken together, our results highlight two complementary sources of disruption in SZ: first, an imbalance in the distribution of dynamic states across key networks, and second, abnormal convergence relationships among states within these systems. Both effects suggest that patients oscillate between extremes of inactivity and hyper-engagement, with reduced representation of intermediate states that support flexible network adaptation. The convergence findings further indicate that not only are states imbalanced in prevalence, but their coordination is also impaired, pointing to instability in how networks transition between functional modes. These results underscore the value of the NeuroMark-DyFICA framework in revealing multilevel abnormalities in brain dynamics that go beyond static connectivity measures, thereby offering new insight into the network-level mechanisms underlying SZ. By focusing on high-frequency spatial variations, NeuroMark-DyFICA captures transient network reconfigurations that relate to flexible cognition and are disrupted in SZ, highlighting the potential clinical relevance of fine-grained spatial dynamics. By applying high-pass filtering after spatially constrained ICA, NeuroMark-DyFICA highlights fine-scale spatial deviations within canonical networks, rather than modifying global network structure, ensuring that subtle pathological variations are captured without altering normative topography.

The fMRI data were preprocessed with a 6 mm FWHM Gaussian kernel to reduce noise and harmonize intersite smoothness. While smoothing improves signal-to-noise ratio, it also attenuates high-frequency spatial details, potentially blurring fine-scale topographic variations. Consequently, the observed high-frequency network dynamics may represent a conservative estimate of true variability. Reducing the smoothing kernel or omitting smoothing could increase sensitivity to subtle spatial shifts but may also amplify noise and site-specific variability, particularly in multisite datasets. Our combination of spatially constrained ICA and high-pass spatial filtering mitigates some smoothing effects, allowing reliable detection of fine-grained dynamic network patterns even after the 6-mm smoothing step. Future studies could systematically investigate the trade-off between smoothing and detection of high-frequency network dynamics to optimize methodological sensitivity.

## LIMITATION

While the NeuroMark-DyFICA framework provides a novel approach for capturing fine-scale spatiotemporal variability, several limitations should be acknowledged. First, the method is specifically optimized to enhance detection of small spatial shifts, as demonstrated in our simulation design and subsequent fMRI analyses. Although this focus is valuable for detecting subtle abnormalities, it may not fully capture larger-scale spatial reorganizations or other forms of dynamic reconfiguration beyond the current scope. Developing a more general framework capable of jointly characterizing both fine-grained and large-scale spatial dynamics represents an important future direction.

Second, the NeuroMark-DyFICA framework relies on spatial priors in its initial decomposition stage. While these priors improve reproducibility and cross-subject correspondence, they may bias estimation toward canonical network configurations derived from healthy populations, potentially reducing sensitivity to rare, novel, or highly individualized spatial patterns. Although subsequent stages of our pipeline—including high-pass spatial filtering and group-level ICA—are explicitly designed to emphasize deviations from these priors, some degree of prior influence remains inherent. Future work could explore adaptive or partially unconstrained extensions of the framework to further balance stability with sensitivity to atypical network organization.

Third, our validation was limited to controlled 2D simulations and a single clinical dataset (SZ). While these provide proof-of-concept evidence, further evaluation on diverse datasets, including different tasks, modalities (e.g., EEG-fMRI), and clinical populations, will be helpful to further assess the robustness and generalizability of the approach.

In addition, external validation using independent datasets would provide a stronger test of reproducibility, but such analyses require substantial computational resources and large-scale data, which were beyond the capacity of the current setup.

Finally, like most sliding-window approaches, parameter choices such as window length and filtering settings may influence the observed dynamics. In this study, we adopted a window length of 60 consecutive time points with partial overlap, following prior work and balancing sensitivity with computational feasibility. Shorter windows could increase temporal resolution but may reduce the reliability of ICA component estimation, while longer windows provide more stable estimates at the cost of smoothing over fast dynamic changes. However, a systematic evaluation of different window lengths is needed to fully assess robustness, particularly since varying the number of windows (e.g., ~40–50 vs. longer segments) may impact sensitivity to temporal dynamics. Future studies could also benefit from multi-scale or adaptive methods, such as filter bank or time-frequency approaches, to capture connectivity fluctuations across a broader range of temporal resolutions.

## CONCLUSION AND FUTURE WORK

In this work, we introduced the NeuroMark-DyFICA framework, a novel approach designed to improve detection of dynamic and fine-scale spatiotemporal variations in fMRI data. By combining spatially constrained ICA, high-pass spatial filtering, and group-level decomposition, the framework captures refined dynamic functional patterns while preserving biologically meaningful network structure. We first validated the method using controlled 2D simulations, which confirmed its ability to detect subtle spatial shifts resembling group-level differences. These results provided a proof-of-concept that windowed ICA combined with spatial filtering can enhance the ability to detect to fine-scale variations that conventional approaches often overlook.

Application to resting-state fMRI data from SZ patients and HCs further demonstrated the utility of the framework. Two complementary analyses—dynamic state density and convergence—revealed recurrent abnormalities across auditory, visual/fusiform, frontal, default mode, and cerebellar networks. Patients exhibited both an imbalance in the distribution of dynamic states, characterized by overrepresentation of inactivity and hyper-engaged states, and altered convergence between specific state pairs, reflecting impaired coordination of network dynamics. These results align with prior reports of network instability and reduced flexibility in SZ, while also extending them by demonstrating the dual impact on both state prevalence and state coordination.

In summary, this study establishes NeuroMark-DyFICA as a conceptually novel and reproducible framework for characterizing spatial chronnectome dynamics—that is, transient, fine-scale reconfigurations of network topography over time. Beyond SZ, the framework is broadly applicable to other neuropsychiatric and neurological conditions, where uncovering subtle spatiotemporal patterns may improve mechanistic understanding and facilitate biomarker development.

In addition, future work should explore extensions of the framework that relax or adapt normative priors, systematically investigate the impact of different spatial smoothing strategies and spatial-frequency bands, as well as more extensive reliability assessments across independent datasets, to further strengthen reproducibility while maintaining sensitivity to atypical network configurations.

Future work should focus on external validation using independent datasets to further confirm reproducibility and generalizability, integration with multimodal data (e.g., EEG-fMRI) to link spatial and temporal dynamics, and, longitudinal studies to track disease progression. In addition, expanding the framework toward multiscale modeling may enable simultaneous characterization of both fine-grained and large-scale dynamics, further bridging the gap between neural mechanisms and clinical translation for precision diagnostics and treatment monitoring.

## ACKNOWLEDGMENTS

The authors acknowledge support for data collection from the National Center for Research Resources of the National Institutes of Health under grants 1 U24 RR021992, 1 U24 RR025736-01, R01EB020407, P20GM103472, and P30GM122734, as well as from the National Science Foundation under grant 1539067.

## AUTHOR CONTRIBUTIONS

Neda Behzadfar: Conceptualization; Formal analysis; Methodology; Software; Validation; Visualization; Writing – original draft; Writing – review & editing. Armin Iraji: Conceptualization; Supervision; Writing – review & editing. Najme Soleimani: Writing – review & editing. Tulay Adali: Data curation; Writing – review & editing. Vince Calhoun: Conceptualization; Data curation; Formal analysis; Funding acquisition; Investigation; Methodology; Project administration; Resources; Software; Supervision; Validation; Visualization; Writing – original draft; Writing – review & editing.

## FUNDING INFORMATION

Vince Calhoun, National Institute of Mental Health and Neurosciences (https://dx.doi.org/10.13039/100019274), Award ID: R01MH129493. Vince Calhoun, Directorate for Education and Human Resources (https://dx.doi.org/10.13039/100000081), Award ID: 2112455.

## DATA AVAILABILITY

The datasets analyzed in this study consist of resting-state fMRI data from 131 patients with SZ and 141 HCs obtained from the Phase III dataset of the Functional Biomedical Informatics Research Network (fBIRN) (Potkin & Ford, 2009). Access to these data is subject to the policies and procedures of the fBIRN data repository. These datasets are not publicly available.

## TECHNICAL TERMS

Spatial chronnectome: Time-varying spatial reorganization of functional brain networks reflecting transient topographic changes.
Dynamic frequency-informed ICA (DyFICA): A dynamic ICA framework enhancing fine-scale spatial fluctuations via sliding windows and spatial frequency filtering“. This definition is consistent with the description provided in the manuscript.
High-pass spatial filtering: Suppression of low spatial-frequency structure to emphasize fine-scale, localized network variations.
High spatial-frequency features: Fine-grained spatial variations capturing subtle, transient network topography beyond large-scale organization.
NeuroMark ICA: A spatially constrained ICA approach using large-sample normative priors for reproducible network estimation.
Latent space of high-frequency dynamics: Hidden fine-scale spatial patterns representing transient reorganization within canonical brain networks.
Spatially constrained ICA: Independent component analysis (ICA) incorporating spatial templates to preserve network correspondence while permitting subject-specific variability.
Dynamic state density: Measure quantifying how frequently specific dynamic spatial states dominate across time windows.
Dynamic convergence and divergence: Assessment of the degree to which dynamic brain state amplitudes align or separate over time.
